# Accessing Care Services for Long COVID Sufferers in Alberta, Canada: A Random, Cross-Sectional Survey Study

**DOI:** 10.3390/ijerph20156457

**Published:** 2023-07-27

**Authors:** Jacqueline A. Krysa, Sidney Horlick, Kiran Pohar Manhas, Katharina Kovacs Burns, Mikayla Buell, Maria J. Santana, Kristine Russell, Elizabeth Papathanassoglou, Chester Ho

**Affiliations:** 1 Neurosciences, Rehabilitation, and Vision Strategic Clinical Network, Alberta Health Services, Edmonton, AB T5J 3E4, Canadamikayla.buell@ucalgary.ca (M.B.); russek14@mcmaster.ca (K.R.);; 2 Division of Physical Medicine and Rehabilitation, Faculty of Medicine and Dentistry, University of Alberta, Edmonton, AB T6G 2E1, Canada; 3 Faculty of Nursing, University of Alberta, Edmonton, AB T6G 1C9, Canada; 4 Community Health Sciences, Cumming School of Medicine, University of Calgary, Calgary, AB T2N 1N4, Canada; 5 School of Public Health, University of Alberta, Edmonton, AB T6G 1C9, Canada; 6 Department of Clinical Quality Metrics, Alberta Health Services, Edmonton, AB T5J 3E4, Canada

**Keywords:** long COVID-19, healthcare navigation, patient experience

## Abstract

Designing appropriate rehabilitation programs for long COVID-19 remains challenging. The purpose of this study was to explore the patient experience of accessing long COVID-19 rehabilitation and recovery services. In this cross-sectional, observational study, a telephone survey was administered to a random sample of persons with long COVID-19 in a Canadian province. Participants included adults who tested positive for COVID-19 between March and October 2021. Survey respondents (*n* = 330) included individuals who had been previously hospitalized for COVID-19 (*n* = 165) and those who had not been hospitalized (‘non-hospitalized’) for COVID-19 (*n* = 165). Significantly more previously hospitalized respondents visited a family doctor for long COVID-19 symptoms compared to non-hospitalized respondents (hospitalized: *n* = 109 (66.1%); non-hospitalized: *n* = 25 (15.2%); (*p* < 0.0001)). Previously hospitalized respondents reported significantly more referrals to specialty healthcare providers for long COVID-19 sym`ptoms (hospitalized: *n* = 45 (27.3%); non-hospitalized: *n* = 6 (3.6%); (*p* < 0.001)). A comparable number of respondents in both groups accessed care services that did not require a referral to manage their long COVID-19 symptoms (hospitalized: *n* = 31 (18.8%); non-hospitalized: *n* = 20 (12.1%); (*p* = 0.20)). These findings demonstrate the diversity of recovery services used by individuals with long COVID-19 and emphasize the need for multidisciplinary long COVID-19 rehabilitation and recovery care pathways.

## 1. Introduction

Long COVID-19 is an emerging condition that describes new or persisting symptoms after recovery from COVID-19 that includes new or lingering symptoms reported during ongoing symptomatic COVID-19 (4–12 weeks since initial recovery) and post-COVID-19 conditions (12 weeks or more since initial recovery) [1]. Long COVID-19 has likely impacted >145 million individuals worldwide [2], with a prevalence varying regionally and between variants of COVID-19 [3,4]. In 2022, the Canadian COVID-19 Antibody and Health Survey found that prior to December 2021 (predominately during the wave of the Alpha and Delta variants of COVID-19) more than one quarter of adults experienced long COVID-19 at least three months after their infection compared to 10.5% of adults after December 2021 (when it was predominately the Omicron variant of COVID-19) [5]. Long COVID-19 represents over 100 diverse symptoms that vary in duration and severity [6,7], with common symptoms including fatigue, headache, dyspnea, mental health concerns, and chest pain [5,6,7]. These symptoms have been shown to significantly impair function, impact quality of life, and interfere with employment and participation in everyday activities [8,9]. Individuals with pre-existing comorbidities and those requiring hospitalization for COVID-19 appear to be at increased risk for long COVID-19 [10,11,12,13,14,15,16]; however, long COVID-19 has been identified in those that experienced a mild initial infection [7,17].

Recovery from COVID-19 has been found to be associated with an increase in healthcare service utilization up to 6 months following diagnosis [18,19] as well as an increased probability and counts of primary and specialty care visits [20]. In an observational cohort study of 64,011 patients, the average number of healthcare visits in the first 30 days after COVID-19 diagnosis was 12.3 for those previously hospitalized and 6.6 for those not hospitalized for COVID-19 [19]. Although medical costs for treating long COVID-19 have not been clearly evaluated [21], annual costs for treating similar post-viral conditions, such as myalgic encephalomyelitis, are estimated annually at USD 9000 per person [22]. Access to high-quality, outpatient care services, including primary and specialty care, is fundamental to supporting the recovery, preventing adverse health outcomes, and reducing overall healthcare costs [23,24,25,26]. This underscores the importance of developing effective and appropriate service models that support the recovery and management of long COVID-19 in the community.

The International G7 Science Ministers meeting in June 2022 prioritized the need to better understand appropriate healthcare and rehabilitation strategies to support the care management of individuals with long COVID-19 [27]. In 2023, the WHO released clinical guidance on the rehabilitation of adults with long COVID-19. Conditional recommendations outlined a need for a multidisciplinary workforce to manage long COVID-19 symptoms, including, but not limited to physiotherapists, occupational therapists, nurses, speech and language therapists, physicians, and social workers [28]. Understanding the types of appropriate rehabilitation and recovery services for long COVID-19 remains challenging [29,30,31]. Considering the complexity of long COVID-19, it is imperative to learn from patients with experience of long COVID-19 to understand the types of rehabilitation programs and services that best support their recovery needs [32,33,34,35,36,37]. For healthcare systems to meet the needs of persons with long COVID-19, they require an in-depth understanding of those with long COVID-19 and their experiences of accessing care support and resources [38,39,40,41].

Studies exploring the patient experience of COVID-19 recovery and service navigation have identified common barriers to long COVID-19 care including the accessibility of long COVID-19 recovery services as well as the inconsistent acknowledgement of symptoms [42,43]. A 2022 qualitative systematic review (*n* = 5 studies) explored the experiences of living with long COVID-19 and accessing healthcare services [43]. This review highlighted the significant impact of long COVID-19 symptoms on quality of life and identified that healthcare provider knowledge of long COVID-19 was a common barrier for persons with long COVID-19 to effectively access and navigate healthcare support [43]. A noted limitation of this review is that participants from all included studies were recruited through social media or online support groups, which the authors noted could enhance the bias of the convenience sample and reduce their likeness to the general population [44,45]. Considering the diversity of symptoms, the impact of previous hospitalization, and the uncertainty of appropriate long COVID-19 rehabilitation services, there is a need to explore the patient experience of COVID-19 care service navigation, including the nuances of recovery needs between those that experienced severe and mild acute COVID-19.

## 2. Materials and Methods

### 2.1. Study Design

This cross-sectional, observational study involved administering a telephone survey to a random sample of adults recovering from COVID-19 to understand their experience of long COVID-19 healthcare access and navigation. This study is part of a broader mixed-methods evaluation study exploring the early implementation of a novel long COVID-19 rehabilitation framework. This study was approved by the University of Alberta Research Ethics Board (Pro00113182). All respondents consented to participate in this study. Reporting was guided by the Checklist for Reporting of Survey Studies (CROSS) (see Supplementary File S1) [46].

### 2.2. Study Population

The study population included adults of ≥18 years with a laboratory-confirmed COVID-19 polymerase chain reaction (PCR) test between March 2021, and October 2021 in Alberta, Canada. Eligible participants had to be able to read and understand English (with or without the assistance of an available family/friend). There were no explicit exclusion criteria.

### 2.3. Sampling

A proportional stratified sampling frame was developed by professional telephone surveys to facilitate recruitment based on COVID-19 hospitalization status (50% hospitalized and 50% non-hospitalized) as well as the primary place of residence (60% metropolitan–urban residence and 40% regional–urban/rural residence) [47]. The study sought to recruit 300 respondents. This number was proposed to allow for representation within each stratum, to support the feasibility and timeliness of results required to inform health system planning, and to account for the limited number of previously hospitalized individuals in Alberta, Canada, during the study period. To further minimize response and non-response bias and ensure a saturation of responses, recruitment continued past the target of 300 respondents.

### 2.4. Survey Design

A novel survey tool was developed to explore common themes derived from a co-design session with provincial stakeholders, including patient and family advisors with lived experience of long COVID-19, clinicians, and administrative, operational, and health system leadership to inform broader health system innovation for long COVID-19 service delivery (see Supplementary File S2) [48]. Key themes included patient and provider knowledge of long COVID-19, the appropriateness of information and referrals, and accessibility of care. These themes were used to inform items of the novel survey. The survey items included closed, multiple-response, and open-ended questions. To identify respondents experiencing long COVID-19 symptoms, one survey item asked, ‘are you experiencing any new or lasting/ongoing symptoms after recovery from COVID-19’. This wording was recommended to avoid confusion about the definition of long COVID-19 from respondents. Survey items were designed to understand general and specific experiences regarding health system access and navigation following COVID-19, as well as open-ended questions on perceived challenges, positive experiences, and improvement suggestions. The use of services required or not required was extracted from the questions: “Which of these specialty healthcare provider(s) were you referred to?” and “What care services did you access without a referral?”, respectively. Both items featured the option ‘select all that apply’ allowing respondents to choose multiple options. Participants were asked between 21 and 37 questions depending on their responses for branching questions.

### 2.5. Recruitment and Data Collection

Eligible hospitalized and non-hospitalized COVID-19 respondents were randomly identified through Alberta Health Services administrative COVID-19 databases by health-system-employed, professionally trained telephone surveyors until the target number of cases and strata was achieved [47]. The use of telephone surveyors was necessary due to privacy regulations at the time, restricting recruitment via mailout or electronic survey. Surveyors provided a brief study introduction and outlined participation requirements over the telephone. Those who agreed to participate provided verbal consent. For individuals that were busy (but agreed to be called back) or did not respond to the initial call, surveyors would make up to three call backs, as needed ensuring all randomly selected individuals had the opportunity to respond or decline the survey. Select surveyors were instructed to administer the survey to interested respondents and recorded responses using VOXCO, a secure and private survey software to prevent unauthorized access (VOXCO Survey Software, Montréal, QC, Canada, 2022).

### 2.6. Data Analysis

Survey data were de-identified by surveyors prior to analysis by the research team. SPSS (IBM SPSS Statistics 25, Chicago, IL, USA, 2022) was used to analyze data. Descriptive statistics were used to describe and summarize the results. ‘Not Applicable’ or ‘Don’t Know’ responses were presented are two additional categories. Comparisons of proportions between groups (including hospitalization status, gender, and geographical location) were examined using Pearson’s Chi-square test of independence. For survey items that had ‘select all that apply’, independent comparisons were conducted. Missing data (either due to omitted answers or ‘not applicable’ answers) were reported for all variables and accounted for in the analysis. Statistical significance was set at *p* < 0.05.

## 3. Results

### 3.1. Participant Demographics

In this study, we contacted 1131 individuals to participate in the survey. Telephone surveyors identified 514 randomly selected individuals that were previously hospitalized from a larger pool of 4728 eligible participants. Of the 514, 222 were disqualified, 112 refused to participate, 15 had indeterminant responses, and 165 completed the survey (59.6% response rate). A group of 617 randomly selected non-hospitalized individuals were identified from a larger pool of 112,449 eligible participants. Of these, 305 were disqualified, 6 had indeterminant responses, 141 refused to participate, and 165 completed the telephone survey (53.92%). The present survey had 330 respondents, including 150 previously hospitalized and 150 non-hospitalized respondents. The completion of the survey took between 7 and 64 min (median 10 min). Table 1 presents the respondent demographics. Previously hospitalized individuals were generally older than non-hospitalized respondents with approximately one-third of previously hospitalized respondents being older than 65 (54 (32.7%)) compared to less than 10% of non-hospitalized individuals (15 (9.1%)). Most respondents were of white ethnicity (106 (64.2%) hospitalized; 124 (75.2%) non-hospitalized, *p* = 0.03). Almost half of previously hospitalized respondents self-reported long COVID-19 symptoms following a positive diagnosis of COVID-19 (81 (49.1%)). This was significantly greater compared to the number of self-reported symptoms by non-hospitalized respondents (42 (25.5%), *p* < 0.0001) [48].

### 3.2. Experience of Access and Appropriateness of Primary Care to Manage COVID-19 Post-Acute Recovery

Significantly more previously hospitalized respondents visited a family doctor after their initial hospitalization to manage their long COVID-19 symptoms compared to non-hospitalized respondents (previously hospitalized: *n* = 109 (66.1%); non-hospitalized: *n* = 25 (15.2%), (*p* < 0.0001)) (Table 2a).

A comparable number of respondents in both groups indicated that their family doctor helped manage their long COVID-19 symptoms (81 hospitalized (49.1%) versus 20 non-hospitalized (80.0%) (*p* = 0.43)). A similar proportion of respondents in both groups also felt that access to their family doctor was timely (88 hospitalized (80.7%) versus 22 non-hospitalized (88.0%) (*p* = 0.39)) (Table 2b).

### 3.3. Experience of Access and Appropriateness of Long COVID-19 Referrals

Previously hospitalized respondents reported significantly more referrals to specialty healthcare providers to help manage their long COVID-19 symptoms compared to non-hospitalized respondents (previously hospitalized: *n* = 45 (27.3%); non-hospitalized: *n* = 6 (3.6%); (*p* < 0.0001)). While waiting to see a healthcare provider, significantly more previously hospitalized respondents had to visit the emergency room or urgent care (previously hospitalized: *n* = 29 (17.6%); non-hospitalized: *n* = 7 (4.2%); (*p* < 0.0001)) (Table 3a).

Table 3b outlines the types of referrals accessed to manage long COVID-19 symptoms as well as the experience of these referrals as self-reported by survey respondents. Compared to the number of non-hospitalized respondents, significantly more previously hospitalized respondents indicated that they received a referral for a physiotherapist (previously hospitalized: *n* = 21 (66.0%); non-hospitalized: *n* = 0 (0.0%); (*p* = 0.03)), respiratory therapist (previously hospitalized: *n* = 24 (53.3%); non-hospitalized: *n* = 0 (0.0%); (*p* = 0.01)), and other types of referrals (previously hospitalized: *n* = 8 (17.8%); non-hospitalized: *n* = 4 (66.7%); (*p* = 0.008)). Significantly more previously hospitalized participants found their referral to be timely (23 (51.1%)) compared to non-hospitalized participants (2 (33.3%) (*p* < 0.0001)). Most respondents in both groups found this referral either to be helpful or somewhat helpful at supporting their COVID-19 recovery (previously hospitalized: *n* = 31 (68.9%); non-hospitalized: *n* = 5 (83.3%); (*p* = 0.61)).

### 3.4. Experience of Access to Non-Referred Services to Support COVID-19 Recovery

A comparable number of respondents in both groups self-reported accessing care services that did not require a referral to support their COVID-19 recovery (previously hospitalized: *n* = 31 (18.8%); non-hospitalized *n* = 20 (12.1%); (*p* = 0.20)) (Table 4a).

Table 4b summarizes the types of services accessed that did not need a referral. Previously hospitalized participants commonly accessed massage therapy (15 (48.4%)), naturopathy (14 (45.2%)), and physiotherapy (9 (29.0%)) services to help manage their symptoms (in comparison to non-hospitalized responders *p* > 0.05). Non-hospitalized participants commonly reported accessing massage therapy (9 (45.0%)), acupuncture (9 (45.0%)), naturopathy (8 (40.0%)) and mental health services (8 (40.0%)) to support their recovery (in comparison to previously hospitalized participants *p* > 0.05).

### 3.5. Experience of Access to Paid or Partially Paid for Services to Support COVID-19 Recovery

Less than half of the previously hospitalized (66 (40.0%)) and one-third of the non-hospitalized respondents (55 (33.3%) (*p* = 0.10)) self-reported having full or private insurance coverage to help pay for the services they accessed to support their COVID-19 recovery. Significantly more previously hospitalized participants self-reported paying or partially paying for the services accessed to manage recovery compared to non-hospitalized respondents (previously hospitalized: *n* = 40 (24.2%); non-hospitalized: *n* = 22 (13.3%); (*p* = 0.03)) (Table 5).

## 4. Discussion

The current study provides novel evidence to highlight the experience and types of services accessed by individuals with long COVID-19 that were previously hospitalized or not hospitalized for COVID-19 between March and October 2021. A previous publication on this survey study reported that significantly more previously hospitalized participants reported long COVID-19 symptoms compared to those not hospitalized [48]. To date, there remains limited evidence on underlying effective and appropriate management strategies for long COVID-19 [13,49,50]. Current recommendations underscore the importance of community-based continuity of care to support the monitoring and treatment of complex and chronic symptoms, with referrals to specialty services when required [26,36,51]. To better support health service planning for COVID-19 recovery and long COVID-19, the present study investigated the experience of long COVID-19 health service access and navigation.

Mounting evidence suggests that pre-existing comorbidities increase the likelihood of developing long COVID-19 [13,14,16,52]. Multimorbidity is strongly associated with hospitalization, with risk increasing with age [53]. Outpatient primary care has been described as appropriate for the management of long COVID-19 symptoms and supporting care transitions from hospital [25,26,54]. In the present study, previously hospitalized respondents were significantly older (*p* < 0.0001), had a greater number of self-reported long COVID-19 symptoms (*p* < 0.0001), and were more likely to visit a family doctor for their long COVID-19 symptoms (hospitalized: *n* = 109 (66.1%); non-hospitalized: *n* = 25 (15.2%); (*p* < 0.0001)). Although there were fewer non-hospitalized respondents visiting a family doctor, most respondents in this group reported that their family doctor helped manage their symptoms compared to less than 50% of the hospitalized group who reported this (*p* = 0.40). Although this was not a significant difference, it may highlight differences in the experience and expectations of a family doctor to manage long COVID-19. A 2020 qualitative study exploring the experiences of primary care in individuals with long COVID-19 revealed that people with long COVID-19 generally experienced limited recognition of their symptoms by family doctors, with many perceiving that healthcare professionals felt their symptoms did not warrant further care [42]. This study emphasized the importance of finding the right family doctor to support the management of long COVID-19 care [42]. In a 2022 review of qualitative studies exploring the experience long COVID-19 and accessing healthcare services, the authors identified that the lack of healthcare provider knowledge on long COVID-19 was as a common barrier to the patient experience of care [43]. While there remains limited knowledge on proven therapies for long COVID-19, there is a need for comprehensive, patient-centered care to support those living with long COVID-19 [55,56]. This emphasizes the need to continue supporting widespread provider education on the identification of and options for long COVID-19 symptoms management [43].

This survey further investigated the types of services accessed by those recovering from COVID-19. Approximately one-third of previously hospitalized participants received a referral compared to less than five percent of non-hospitalized participants (*p* < 0.0001). Compared to the number of non-hospitalized respondents, significantly more previously hospitalized respondents indicated they received a referral for a physiotherapist (*p* = 0.03), and respiratory therapist (*p* = 0.01). Emerging evidence suggests a role in COVID-19 recovery and increased health service utilization [56]. In a 2021 population-based study monitoring healthcare utilization following COVID-19, it was found that after recovery from acute infection, approximately 40% of individuals had at least contact with a healthcare professional which was related to COVID-19 [56]. A 2022 publication reported that healthcare utilization was twice as high for previously hospitalized individuals compared to non-hospitalized individuals up to 6 months after a COVID-19 diagnosis [19]. This evidence highlights the burden of COVID-19 recovery and long COVID-19 on health service utilization and stresses the need for the appropriate and timely management of symptoms.

Timeliness of care has been identified as a common patient concern in other studies exploring the long COVID-19 patient experience of care [43,55]. Almost half of previously hospitalized respondents agreed that their referral to a specialty provider was timely and this was a significantly greater proportion compared to that of non-hospitalized participants (hospitalized: *n* = 23 (51.1%); non-hospitalized: *n* = 2 (33.3%); (*p* < 0.0001)). Despite the perception of timeliness, a significant number of previously hospitalized respondents reported needing to visit the emergency room or urgent care center while waiting to see a healthcare provider (17.6% previously hospitalized versus 4.2% non-hospitalized, *p* < 0.0001). In a 2023 survey study, individuals with long COVID-19 expressed a desire for better and more rapid access to appointments with specialists to manage their symptoms [55]. Timely outpatient care following discharge from hospital is paramount to enhancing care coordination and preventing hospital readmissions [57,58]. Findings from several observational studies have found that patients that receive outpatient follow-up care have a significantly lower risk of 30-day readmissions [59,60,61,62]. These findings stress the need for enhanced continuity of care for persons recovering from COVID-19, especially for those that have been previously hospitalized.

A comparable number of respondents in both groups indicated accessing services that did not require a referral (previously hospitalized: *n* = 31 (18.8%); non-hospitalized: *n* = 20 (12.1%); (*p* = 0.20)). Common services accessed without a referral included acupuncture (*p* = 0.83), chiropractic care (*p* = 0.29), and massage therapy (*p* = 0.81). Similar to other post-viral syndromes, it has been proposed that long COVID-19 requires an integrated healthcare approach, including traditional management, non-pharmacological approaches, and behavior and lifestyle changes to manage the wide-spread impact of symptoms on individual physical, mental, behavioral, social and spiritual health [55,63]. A 2023 crowdsourced research study of approximately 500 individuals with self-reported long COVID-19 identified that mind–body medicine and respiratory therapy were common and helpful symptom management approaches [55]. Growing evidence also underscores the need for increased mental health support for those recovering from COVID-19 and from the long-term impacts of social isolation from the pandemic [64]. Recognizing this need for multidisciplinary care, countries including Canada [65,66], the United States of America [67], and United Kingdom [68,69] have established specialty long COVID-19 clinics, offering specialized, multidisciplinary outpatient medical and rehabilitation care support for individuals recovering from long COVID-19 in the community.

More than half of previously hospitalized and non-hospitalized respondents in the present study noted that they did not have full or private insurance coverage for the services they accessed to manage their long COVID-19 (*p* = 0.10). Financial burden of care is a widespread issue and is especially significant in those with a multimorbidity, that are noted to have between five- and ten-fold-higher out of pocket costs compared to those without pre-existing conditions [70,71]. A 2010 study found that individuals with financial concerns were more likely to delay seeking care [72]. The present study did not investigate individual socioeconomic status, which may limit our understanding of how financial strain impacts healthcare navigation and long COVID-19.

The strengths and limitations of this study have been described in more detail here [48]. The present study design was strengthened by the robust, stratified, random survey sampling approach used by professional telephone surveyors. This allowed for a representative population to be surveyed, for a direct comparison between hospitalized and non-hospitalized groups and for the better representation of the general population. This study was conducted between April 2022 and June 2022, which provided between 6 months to 13 months of follow-up allowing time to better understand the experience with service navigation to support COVID-19 recovery and long COVID-19.

Notable limitations of the study include the use of a novel, co-designed survey tool, which was necessarily developed to explore the novelty of experience of health service navigation for long COVID-19. This tool has not been validated, possibility impacting the study’s comparability to similar studies [73,74]. The development of a novel survey tool was necessary to explore priority themes from the co-design session to inform health system planning and due to the limited availability of relevant survey tools at the time of this study. However, the use of cognitive interviews allowed for preliminary assessments of survey content by persons with lived experience of long COVID-19. The present survey study included individuals that tested positive for COVID-19 between March 2021, and October 2021. This may have limited applicability to those that experienced the Omicron variant of COVID-19, which has been found to result in differing long COVID-19 symptomology than earlier variants [75,76]. Additionally, this study did not capture whether or not this experience was a participant’s first or subsequent COVID-19 infection, which could negatively impair recovery and impact the individual experience of recovery. This study also relied on the self-reporting of long COVID-19 symptoms due to the limited screening and assessment tools available to accurately diagnose long COVID-19 at the time of data collection [77]. Participant vaccination status was not collected due to data restrictions at the time of this study, which restricted inferences regarding vaccination status and the experience of long COVID-19. Although the present survey collected data on individual locations of residence, it did not capture information on individual socioeconomic status, anthropometrics, pre-existing conditions, or lifestyle habits which have an impact and result in significant disparities in COVID-19 recovery, quality of life, and overall healthcare utilization [13,15,78,79]. Future studies should further investigate experiences of healthcare access for COVID-19 recovery in vulnerable populations as well as individuals with comorbidities to better elucidate the specific resources and rehabilitation and lifestyle strategies required to manage long COVID-19 symptoms in these higher-risk sub-populations.

## 5. Conclusions

The experience of long COVID-19 is varied and often challenging for those seeking acknowledgement of their symptoms and effective care management [43]. This study suggests that individuals with both mild and severe long COVID-19 symptoms seek out a diversity of services to manage their symptoms and reveals that wait times and costs of care remain a barrier to access. This supports the need for personalized, multidisciplinary rehabilitation support for individuals recovering from COVID-19 in the community.

## Figures and Tables

**Table 1:** Survey participant demographics by hospitalization status.

Item	Hospitalized (*n* = 165)	Non-Hospitalized (*n* = 165)	*p* Value χ2
Age Group (Years)			<0.0001
18–40	23 (13.9%)	77 (46.7%)	
41–65	88 (53.3%)	72 (43.6%)	
>65	54 (32.7%)	15 (9.1%)	
Refused	0 (0.0%)	1 (0.6%)	
Female Gender	72 (43.6%)	73 (44.2%)	0.99
Metropolitan-Urban Residence	99 (60%)	99 (60%)	1.00
Racial/Ethnic Groups *			
Caucasian	106 (64.2%)	124 (75.2%)	0.03
Filipino	11 (6.7%)	8 (4.8%)	0.48
Indigenous	8 (4.8%)	6 (3.6%)	0.56
South Asian	7 (4.2%)	9 (5.5%)	0.61
Other	35 (21.2%)	30 (18.1%)	0.09
Are you having new or lasting/ongoing symptoms since you first tested positive for COVID-19?			
Yes	81 (49.1%)	42 (25.5%)	<0.0001
No	78 (47.3%)	118 (71.5%)	
Don’t Know	6 (3.6%)	5 (3.0%)	

* Participants were invited to select all that apply, therefore, values will not add up to 100%.

**Table 2:** (**a**) Experience of access and appropriateness of primary care to manage long COVID-19. (**b**) Experience of access and appropriateness of family doctor to manage long COVID-19.

(a)				
Item	Hospitalized (*n* = 165)	Item	Non-Hospitalized (*n* = 165)	*p* Value χ^2^
After leaving the hospital, did you see your family doctor because of new and/or lasting symptoms during your recovery from COVID-19?		Did you see your family doctor because of new and/or lasting symptoms during your recovery from COVID-19?		
Yes	109 (66.1%)	Yes	25 (15.2%)	<0.0001
No	56 (33.9%)	No	137 (83.0%)	
Don’t Know		Don’t Know	3 (1.8%)	
**(b)**				
**Item**		**Hospitalized** **(*n* = 109)**	**Non-Hospitalized** **(*n* = 25)**	***p* Value** **χ^2^**
Did your family doctor help you manage your new and/or lasting symptoms?				
Yes		81 (49.1%)	20 (80.0%)	0.43
No		21 (12.7%)	5 (20.0%)	
Don’t Know		7 (4.2%)	0 (0%)	
Did you feel you had timely access to your family doctor to help you manage your new and/or lasting symptoms?				
Yes		88 (80.7%)	22 (88.0%)	0.39
No		21 (19.3%)	3 (12.0%)	

**Table 3:** (**a**) Experience of access and appropriateness of long COVID-19 referrals. (**b**) Experience of access and appropriateness of long COVID-19 referrals.

(a)			
Item	Hospitalized (*n* = 165)	Non-Hospitalized (*n* = 165)	*p* Value χ^2^
Did you need to visit the emergency room or urgent care while waiting to see a healthcare provider for your new or lasting symptoms from COVID-19?			
Yes	29 (17.6%)	7 (4.2%)	<0.0001
No	134 (81.2%)	158 (95.8%)	
Don’t Know	2 (1.2%)	0 (0%)	
Were you referred to specialty healthcare providers to help manage your new and/or lasting symptoms?			
Yes	45 (27.3%)	6 (3.6%)	<0.0001
No	118 (71.5%)	158 (95.8%)	
Don’t Know	2 (1.2%)	1 (0.6%)	
**(b)**			
**Item**	**Hospitalized** **(*n* = 45)**	**Non-Hospitalized** **(*n* = 6)**	***p* Value** **χ^2^**
Which of these specialty healthcare provider(s) were you referred to? *			
Physiotherapists	21 (66.0%)	0 (0.0%)	0.03
Occupational therapist	11 (24.4%)	1 (16.7%)	0.67
Respiratory therapist	24 (53.3%)	0 (0.0%)	0.01
Mental health professional	4 (8.9%)	1 (16.7%)	0.55
Social worker	7 (15.6%)	0 (0.0%)	0.23
Specialist clinic	21 (46.6%)	1 (16.7%)	0.16
COVID-19 clinic specialist	12 (26.7%)	1 (16.7%)	0.60
Other	8 (17.8%)	4 (66.7%)	0.008
Don’t Know	1 (2.2%)	0 (0.0%)	0.71
Was your access to a referred specialty healthcare provider(s) timely?			
Yes	23 (51.1%)	2 (33.3%)	<0.0001
No	20 (44.4%)	4 (66.7%)	
Don’t Know	2 (4.4%)	0 (0.0%)	
Did the referral/referrals help you with your recovery following COVID-19?			
Yes	21 (46.7%)	2 (33.3%)	0.61
Somewhat	10 (22.2%)	3 (50.0%)	
No	11 (24.4%)	1 (16.7%)	
Don’t Know	3 (6.7%)	0 (0.0%)	

* Participants were invited to select all that apply, therefore, values will not add up to 100%.

**Table 4:** (**a**) Experience of access to non-referred services to support long COVID-19. (**b**) Experience of access to non-referred services to support long COVID-19.

(a)			
Item	Hospitalized (*n* = 165)	Non-Hospitalized (*n* = 165)	*p* Value χ^2^
Did you access any other care services that did not need a referral?			
Yes	31 (18.8%)	20 (12.1%)	0.20
No	132 (80.0%)	144 (87.3%)	
Don’t Know	2 (1.2%)	1 (0.6%)	
**(b)**			
**Item**	**Hospitalized** **(*n* = 31)**	**Non-Hospitalized** **(*n* = 20)**	***p* Value** **χ^2^**
What care services did you access without a referral? *			
Physiotherapy	9 (29.0%)	4 (20.0%)	0.47
Mental health	7 (22.6%)	8 (40.0%)	0.18
Massage therapy	15 (48.4%)	9 (45.0%)	0.81
Acupuncture	4 (12.9%)	9 (45.0%)	0.83
Chiropractor	8 (25.8%)	3 (15.0%)	0.29
Naturopathy	14 (45.2%)	8 (40.0%)	0.39
Other	6 (19.4%)	6 (30.0%)	0.09
Don’t know	2 (6.5%)	1 (5.0%)	0.67
Refused	1 (3.2%)	2 (10.0%)	0.69

* Participants were invited to select all that apply, therefore, values will not add up to 100%.

**Table 5:** Experience of access to paid or partially paid for services to support long COVID-19.

Item	Hospitalized (*n* = 31)	Non-Hospitalized (*n* = 20)	*p* Value χ^2^
Did you have full or partial private insurance coverage for any of the services you accessed?			
Yes	66 (40.0%)	55 (33.3%)	0.10
No	89 (53.9%)	87 (52.7%)	
Don’t Know	8 (4.8%)	20 (12.1%)	
Refused	2 (1.2%)	3 (1.8%)	
Did you have to pay or partially pay out-of-pocket for some or any of the services you accessed?			
Yes	40 (24.2%)	22 (13.3%)	0.03
No	121 (73.3%)	134 (81.2%)	
Don’t Know	2 (1.2%)	7 (4.2%)	
Refused	2 (1.2%)	2 (1.2%)	

## Data Availability

The data presented in this study are available on request from the corresponding author. The data are not publicly available due to participant confidentiality.

## Supplementary Materials

The following supporting information can be downloaded at https://www.mdpi.com/article/10.3390/ijerph20156457/s1. File S1: CROSS checklist; File S2: Accessing Care and Services After COVID-19: A Patient Experience Survey.

## References

[1] National Institute for Health and Care Excellence (NICE) COVID-19 Rapid Guideline: Managing the Long Term Effects of COVID-19. https://www.nice.org.uk/guidance/ng188/resources/covid19-rapid-guideline-managing-the-longterm-effects-of-covid19-pdf-51035515742.

[2] Hanson S.W., Abbafati C., Aerts J.G., Al-Aly Z., Ashbaugh C., Ballouz T., Blyuss O., Bobkova P., Bonsel G., Borzakova S. (2022). A global systematic analysis of the occurrence, severity, and recovery pattern of long COVID in 2020 and 2021. medRxiv.

[3] Pavli A., Theodoridou M., Maltezou H.C. (2021). Post-COVID Syndrome: Incidence, Clinical Spectrum, and Challenges for Primary Healthcare Professionals. Arch. Med. Res..

[4] Wiersinga W.J., Rhodes A., Cheng A.C., Peacock S.J., Prescott H.C. (2020). Pathophysiology, Transmission, Diagnosis, and Treatment of Coronavirus Disease 2019 (COVID-19): A Review. JAMA.

[5] Statistics Canada Long-Term Symptoms in Canadian Adults Who Tested Positive for COVID-19 or Suspected an Infection, January 2020 to August 2022. https://www150.statcan.gc.ca/n1/daily-quotidien/221017/dq221017b-eng.htm.

[6] Callard F., Perego E. (2021). How and why patients made Long Covid. Soc. Sci. Med..

[7] Higgins V., Sohaei D., Diamandis E.P., Prassas I. (2020). COVID-19: From an acute to chronic disease? Potential long-term health consequences. Crit. Rev. Clin. Lab. Sci..

[8] Tabacof L., Tosto-Mancuso J., Wood J., Cortes M., Kontorovich A., McCarthy D., Rizk D., Rozanski G., Breyman E., Nasr L. (2021). Post-acute COVID-19 Syndrome Negatively Impacts Physical Function, Cognitive Function, Health-Related Quality of Life, and Participation. Am. J. Phys. Med. Rehabil..

[9] Brehon K., Niemeläinen R., Hall M., Bostick G.P., Brown C.A., Wieler M., Gross D.P. (2022). Return-to-Work Following Occupational Rehabilitation for Long COVID: Descriptive Cohort Study. JMIR Rehabil. Assist. Technol..

[10] O’Mahoney L.L., Routen A., Gillies C., Ekezie W., Welford A., Zhang A., Karamchandani U., Simms-Williams N., Cassambai S., Ardavani A. (2023). The prevalence and long-term health effects of Long Covid among hospitalised and non-hospitalised populations: A systematic review and meta-analysis. eClinicalMedicine.

[11] Pérez-González A., Araújo-Ameijeiras A., Fernández-Villar A., Crespo M., Poveda E. (2022). Long COVID in hospitalized and non-hospitalized patients in a large cohort in Northwest Spain, a prospective cohort study. Sci. Rep..

[12] Katsarou M.-S., Iasonidou E., Osarogue A., Kalafatis E., Stefanatou M., Pappa S., Gatzonis S., Verentzioti A., Gounopoulos P., Demponeras C. (2022). The Greek Collaborative Long COVID Study: Non-Hospitalized and Hospitalized Patients Share Similar Symptom Patterns. J. Pers. Med..

[13] Russell C.D., Lone N.I., Baillie J.A.K. (2023). Comorbidities, multimorbidity and COVID-19. Nat. Med..

[14] Evans R.A., McAuley H., Harrison E.M., Shikotra A., Singapuri A., Sereno M., Elneima O., Docherty A.B., Lone N.I., Leavy O.C. (2021). Physical, cognitive, and mental health impacts of COVID-19 after hospitalisation (PHOSP-COVID): A UK multicentre, prospective cohort study. Lancet Respir. Med..

[15] Subramanian A., Nirantharakumar K., Hughes S., Myles P., Williams T., Gokhale K.M., Taverner T., Chandan J.S., Brown K., Simms-Williams N. (2022). Symptoms and risk factors for long COVID in non-hospitalized adults. Nat. Med..

[16] Xie Y., Bowe B., Al-Aly Z. (2021). Burdens of post-acute sequelae of COVID-19 by severity of acute infection, demographics and health status. Nat. Commun..

[17] Rajan S., Khunti K., Alwan N., Steves C., MacDermott N., Morsella A., Angulo E., Winkelmann J., Bryndová L., Fronteira I. In the wake of the pandemic: Preparing for Long COVID. http://www.euro.who.int/en/about-us/partners/.

[18] Hernandez-Romieu A.C., Leung S., Mbanya A., Jackson B.R., Cope J.R., Bushman D., Dixon M., Brown J., McLeod T., Saydah S. (2021). Health Care Utilization and Clinical Characteristics of Nonhospitalized Adults in an Integrated Health Care System 28–180 Days After COVID-19 Diagnosis—Georgia, May 2020–March 2021. MMWR Morb. Mortal. Wkly. Rep..

[19] Huang B.Z., Creekmur B., Yoo M.S., Broder B., Sharp A.L. (2022). Healthcare Utilization Among Patients Diagnosed with COVID-19 in a Large Inte-grated Health System. J. Gen. Intern. Med..

[20] Roth S.E., Govier D.J., Marsi K., Cohen-Cline H. (2022). Differences in Outpatient Health Care Utilization 12 Months after COVID-19 Infection by Race/Ethnicity and Community Social Vulnerability. Int. J. Environ. Res. Public Health.

[21] Cutler D.M. (2022). The Costs of Long COVID. JAMA Health Forum.

[22] Jason L., Mirin A. (2021). Updating the National Academy of Medicine ME/CFS prevalence and economic impact figures to account for population growth and inflation. Fatigue Biomed. Health Behav..

[23] Sanderson C., Dixon J. (2000). Conditions for Which Onset or Hospital Admission is Potentially Preventable by Timely and Effective Ambulatory Care. J. Health Serv. Res. Policy.

[24] Johnston K.J., Wen H., Maddox K.E.J. (2019). Lack Of Access To Specialists Associated With Mortality And Preventable Hospitalizations Of Rural Medicare Beneficiaries. Health Aff..

[25] Berger Z., DE Jesus V.A., Assoumou S.A., Greenhalgh T. (2021). Long COVID and Health Inequities: The Role of Primary Care. Milbank Q..

[26] Greenhalgh T., Knight M., A’court C., Buxton M., Husain L. (2020). Management of post-acute COVID-19 in primary care. BMJ.

[27] G7 Science Ministers’ Meeting, 12–14 June 2022: Communiqué—GOV.UK. https://www.gov.uk/government/publications/g7-science-ministers-meeting-12-14-june-2022-communique.

[28] Clinical Management of COVID-19: Living Guideline, 13 January 2023. https://www.who.int/publications/i/item/WHO-2019-nCoV-clinical-2023.1.

[29] Herrera J.E., Niehaus W.N., Whiteson J., Azola A., Baratta J.M., Fleming T.K., Kim S.Y., Naqvi H., Sampsel S., Silver J.A.K. (2021). Multidisciplinary collaborative consensus guidance statement on the assessment and treatment of fatigue in postacute sequelae of SARS-CoV-2 infection (PASC) patients. PM R..

[30] Negrini S., Borg K., Cusick A., Ferriero G., Frontera W.R., Gross D.P., Heinemann A., Machalicek W., Moore A.P., Nudo R.J. (2022). Global statements to produce and implement evidence in the post-COVID-19 era provide a path forward for rehabilitation—A joint initiative of Cochrane Rehabilitation and the leading journals in the field. J. Occup. Rehabil..

[31] Décary S., De Groote W., Arienti C., Kiekens C., Boldrini P., Lazzarini S.G., Dugas M., Stefan T., Langlois L., Daigle F. (2022). Scoping review of rehabilitation care models for post COVID-19 condition. Bull. World Health Organ..

[32] Doyle C., Lennox L., Bell D. (2013). A systematic review of evidence on the links between patient experience and clinical safety and effectiveness. BMJ Open.

[33] Wong E., Mavondo F., Fisher J. (2020). Patient feedback to improve quality of patient-centred care in public hospitals: A system-atic review of the evidence. BMC Health Serv. Res..

[34] O’Connell P., Henderson I., Steward S., Ho C. (2021). Provincial Post COVID-19 Rehabilitation Response Framework Summary Report.

[35] Sheehy L.M. (2020). Considerations for Post Acute Rehabilitation for Survivors of COVID-19. JMIR Public. Health Surveill..

[36] Barker-Davies R.M., O’Sullivan O., Senaratne K.P.P., Baker P., Cranley M., Dharm-Datta S., Ellis H., Goodall D., Gough M., Lewis S. (2020). The Stanford Hall consensus statement for post-COVID-19 rehabilitation. Br. J. Sports Med..

[37] Phillips M., Turner-Stokes L., Wade D., Walton K. (2020). Rehabilitation in the wake of COVID-19—A phoenix from the ashes. Br. Soc. Rehabil. Med..

[38] Price R.A., Elliott M.N., Zaslavsky A.M., Hays R.D., Lehrman W.G., Rybowski L., Edgman-Levitan S., Cleary P.D. (2014). Examining the Role of Patient Experience Surveys in Measuring Health Care Quality. Med. Care Res. Rev..

[39] Browne K., Roseman D., Shaller D., Edgman-Levitan S. (2010). analysis & commentary Measuring Patient Experience As A Strategy For Improving Primary Care. Health Aff..

[40] Friedberg M.W., SteelFisher G.K., Karp M., Schneider E.C. (2011). Physician Groups’ Use of Data from Patient Experience Surveys. J. Gen. Intern. Med..

[41] Luxford K. (2012). What does the patient know about quality?. Int. J. Qual. Health Care.

[42] Kingstone T., Taylor A.K., O’Donnell C.A., Atherton H., Blane D.N., Chew-Graham C.A. (2020). Finding the ‘right’ GP: A qualitative study of the experiences of people with long-COVID. BJGP Open.

[43] Macpherson K., Cooper K., Harbour J., Mahal D., Miller C., Nairn M. (2022). Experiences of living with long COVID and of accessing healthcare services: A qualitative systematic review. BMJ Open.

[44] Blank G., Lutz C. (2017). Representativeness of Social Media in Great Britain: Investigating Facebook, LinkedIn, Twitter, Pinterest, Google+, and Instagram. Am. Behav. Sci..

[45] Dolatabadi E., Moyano D., Bales M., Spasojevic S., Bhambhoria R., Bhatti J., Debnath S., Hoell N., Li X., Leng C. (2022). Using Social Media to Help Understand Long COVID Patient Reported Health Out-comes: A Natural Language Processing Approach. medRxiv.

[46] Sharma A., Duc N.T.M., Thang T.L.L., Nam N.H., Ng S.J., Abbas K.S., Huy N.T., Marušić A., Paul C.L., Kwok J. (2021). A Consensus-Based Checklist for Reporting of Survey Studies (CROSS). J. Gen. Intern. Med..

[47] Alberta Health Services AHS Public Surveys. https://www.albertahealthservices.ca/about/page13181.aspx.

[48] Krysa J.A., Buell M., Manhas K.P., Burns K.K., Santana M.J., Horlick S., Russell K., Papathanassoglou E., Ho C. (2023). Understanding the Experience of Long COVID Symptoms in Hospitalized and Non-Hospitalized Individuals: A Random, Cross-Sectional Survey Study. Healthcare.

[49] Sudre C.H., Murray B., Varsavsky T., Graham M.S., Penfold R.S., Bowyer R.C., Pujol J.C., Klaser K., Antonelli M., Canas L.S. (2021). Attributes and Predictors of Long COVID. Nat. Med..

[50] Brennan A., Broughan J., McCombe G., Brennan J., Collins C., Fawsitt R., Gallagher J., Guérandel A., O’Kelly B., Quinlan D. (2022). Enhancing the management of long COVID in general practice: A scoping review. BJGP Open.

[51] O’Brien H., Tracey M.J., Ottewill C., O’brien M.E., Morgan R.K., Costello R.W., Gunaratnam C., Ryan D., McElvaney N.G., McConkey S.J. (2021). An integrated multidisciplinary model of COVID-19 recovery care. Ir. J. Med. Sci..

[52] Office for National Statistics Prevalence of Ongoing Symptoms Following Coronavirus (COVID-19) Infection in the UK—Office for National Statistics. https://www.ons.gov.uk/peoplepopulationandcommunity/healthandsocialcare/conditionsanddiseases/bulletins/prevalenceofongoingsymptomsfollowingcoronaviruscovid19infectionintheuk/7july2022.

[53] Gruneir A., Bronskill S.E., Maxwell C.J., Bai Y.Q., Kone A.J., Thavorn K., Petrosyan Y., Calzavara A., Wodchis W.P. (2016). The association between multimorbidity and hospitalization is modified by individual demographics and physician continuity of care: A retrospective cohort study. BMC Health Serv. Res..

[54] Brooke B.S., Stone D.H., Cronenwett J.L., Nolan B., DeMartino R.R., MacKenzie T.A., Goodman D.C., Goodney P.P. (2014). Early primary care provider follow-up and readmission after high-risk surgery. JAMA Surg..

[55] Klocke C., Valentini J., Stolz R., Gaßner L., Joos S., Förster C. (2023). Patients’ Experiences With Therapeutic Approaches for Post-COVID Syndrome: Results of a Crowdsourced Research Survey. Ann. Fam. Med..

[56] Menges D., Ballouz T., Anagnostopoulos A., Aschmann H.E., Domenghino A., Fehr J.S., Puhan M.A. (2021). Burden of post-COVID-19 syndrome and implications for healthcare service planning: A population-based cohort study. PLoS ONE.

[57] Kim C.S., Flanders S.A. (2013). In the Clinic. Transitions of care. Ann. Intern. Med..

[58] Kripalani S., Theobald C.N., Anctil B., Vasilevskis E.E. (2014). Reducing Hospital Readmission: Current Strategies and Future Directions. Annu. Rev. Med..

[59] Sin D.D., Bell N.R., Svenson L.W., Man S. (2002). The impact of follow-up physician visits on emergency readmissions for patients with asthma and chronic obstructive pulmonary disease: A population-based study. Am. J. Med..

[60] Hernandez A.F., Greiner M.A., Fonarow G.C., Hammill B.G., Heidenreich P.A., Yancy C.W., Peterson E.D., Curtis L.H. (2010). Relationship Between Early Physician Follow-up and 30-Day Readmission Among Medicare Beneficiaries Hospitalized for Heart Failure. JAMA.

[61] Sharma G., Kuo Y.-F., Freeman J.L., Zhang D.D., Goodwin J.S. (2010). Outpatient Follow-up Visit and 30-Day Emergency Department Visit and Readmission in Patients Hospitalized for Chronic Obstructive Pulmonary Disease. Arch. Intern. Med..

[62] Baker H., Oliver-McNeil S., Deng L., Hummel S.L. (2015). Regional Hospital Collaboration and Outcomes in Medicare Heart Failure Patients: See You in 7. JACC Heart Fail..

[63] Roth A., Chan P.S., Jonas W. (2021). Addressing the Long COVID Crisis: Integrative Health and Long COVID. Glob. Adv. Health Med..

[64] Wong S.Y.S., Zhang D., Sit R.W.S., Yip B.H.K., Chung R.Y., Wong C.K.M., Chan D.C.C., Sun W., Kwok K.O., Mercer S.W. (2020). Impact of COVID-19 on loneliness, mental health, and health service utilisation: A prospective cohort study of older adults with multimorbidity in primary care. Br. J. Gen. Pract..

[65] Alberta Health Services Long COVID-19 Inter-Professional Outpatient Program (IPOP): FAQ for Healthcare Providers. https://www.albertahealthservices.ca/assets/info/ppih/if-ppih-covid-19-ipop-faq.pdf.

[66] CIUSSS West-Central Montreal Referral Clinic for Long COVID and Lyme Disease. https://www.ciussswestcentral.ca/zone-for-partners/referral-clinic-for-long-covid-and-lyme-disease/.

[67] Survivor Corps Post-COVID Care: Guidelines for Multidisciplinary Care Centers. https://static1.squarespace.com/static/5e8b5f63562c031c16e36a93/t/605a8a3262f0191b99584df0/1616546355297/PCCC+Standard+of+Practice+3_23.pdf.

[68] NHS England » NHS to Offer ‘Long Covid’ Sufferers Help at Specialist Centres. https://www.england.nhs.uk/2020/10/nhs-to-offer-long-covid-help/.

[69] NHS England Long COVID Patients to Get Help at More than 60 Clinics. https://www.england.nhs.uk/2020/12/long-covid-patients-to-get-help-at-more-than-60-clinics/.

[70] Larkin J., Foley L., Smith S.M., Harrington P., Clyne B. (2020). The experience of financial burden for people with multimorbidity: A systematic review of qualitative research. Health Expect..

[71] Sum G., Hone T., Atun R., Millett C., Suhrcke M., Mahal A., Koh G.C.-H., Lee J.T. (2018). Multimorbidity and out-of-pocket expenditure on medicines: A systematic review. BMJ Glob. Health.

[72] Smolderen K.G., Spertus J.A., Nallamothu B.K., Krumholz H.M., Tang F., Ross J.S., Ting H.H., Alexander K.P., Rathore S.S., Chan P.S. (2010). Health Care Insurance, Financial Concerns in Accessing Care, and Delays to Hospital Presentation in Acute Myocardial Infarction. JAMA.

[73] Dima A.L. (2018). Scale validation in applied health research: Tutorial for a 6-step R-based psychometrics protocol. Health Psychol. Behav. Med..

[74] Hawkins R.J., Swanson B., Kremer M.J., Fogg L. (2014). Content Validity Testing of Questions for a Patient Satisfaction With General Anesthesia Care Instrument. J. PeriAnesthesia Nurs..

[75] Hernández-Aceituno A., García-Hernández A., Larumbe-Zabala E. (2023). COVID-19 long-term sequelae: Omicron versus Alpha and Delta variants. Infect. Dis. Now..

[76] Du M., Ma Y., Deng J., Liu M., Liu J. (2022). Comparison of Long COVID-19 Caused by Different SARS-CoV-2 Strains: A Systematic Review and Meta-Analysis. Int. J. Environ. Res. Public Health.

[77] Groff D., Sun A., Ssentongo A.E., Ba D.M., Parsons N., Poudel G.R., Lekoubou A., Oh J.S., Ericson J.E., Ssentongo P. (2021). Short-term and Long-term Rates of Postacute Sequelae of SARS-CoV-2 Infection. JAMA Netw. Open.

[78] Magesh S., John D., Li W.T., Li Y., Mattingly-App A., Jain S., Chang E.Y., Ongkeko W.M. (2021). Disparities in COVID-19 Outcomes by Race, Ethnicity, and Socioeconomic Status: A Systematic Review and Meta-analysis. JAMA Netw. Open.

[79] Suyanto S., Kandel S., Kemal R.A., Arfianti A. (2022). The Quality of Life of Coronavirus Disease Survivors Living in Rural and Urban Area of Riau Province, Indonesia. Infect. Dis. Rep..

